# Quercetin Exerts Age-Dependent Beneficial Effects on Blood Pressure and Vascular Function, But Is Inefficient in Preventing Myocardial Ischemia-Reperfusion Injury in Zucker Diabetic Fatty Rats

**DOI:** 10.3390/molecules25010187

**Published:** 2020-01-02

**Authors:** Kristina Ferenczyova, Barbora Kalocayova, Lucia Kindernay, Marek Jelemensky, Peter Balis, Andrea Berenyiova, Anna Zemancikova, Veronika Farkasova, Matus Sykora, Lubomira Tothova, Tomas Jasenovec, Jana Radosinska, Jozef Torok, Sona Cacanyiova, Miroslav Barancik, Monika Bartekova

**Affiliations:** 1 Institute for Heart Research, Centre of Experimental Medicine, Slovak Academy of Sciences, 84104 Bratislava, Slovakia; kristina.ferenczyova@savba.sk (K.F.); barbora.kalocayova@savba.sk (B.K.); lucia.griecsova@savba.sk (L.K.); marek.jelemensky@savba.sk (M.J.); weroro@gmail.com (V.F.); matus.sykora@savba.sk (M.S.); jana.radosinska@fmed.uniba.sk (J.R.); Miroslav.Barancik@savba.sk (M.B.); 2 Institute of Normal and Pathological Physiology, Centre of Experimental Medicine, Slovak Academy of Sciences, 81371 Bratislava, Slovakia; piotr.balis@gmail.com (P.B.); andrea.berenyiova@savba.sk (A.B.); anna.zemancikova@savba.sk (A.Z.); Jozef.Torok@savba.sk (J.T.); Sona.cacanyiova@savba.sk (S.C.); 3 Institute of Molecular Biomedicine, Faculty of Medicine, Comenius University in Bratislava, 81372 Bratislava, Slovakia; tothova.lubomira@gmail.com; 4 Institute of Physiology, Faculty of Medicine, Comenius University in Bratislava, 81372 Bratislava, Slovakia; tomas.jasenovec@fmed.uniba.sk

**Keywords:** quercetin, cardiovascular disease, blood pressure, vascular relaxation, ischemia-reperfusion injury, infarct size, RISK pathway

## Abstract

*Background*: Quercetin (QCT) was shown to exert beneficial cardiovascular effects in young healthy animals. The aim of the present study was to determine cardiovascular benefits of QCT in older, 6-month and 1-year-old Zucker diabetic fatty (ZDF) rats (model of type 2 diabetes). *Methods*: Lean (fa/+) and obese (fa/fa) ZDF rats of both ages were treated with QCT for 6 weeks (20 mg/kg/day). Isolated hearts were exposed to ischemia-reperfusion (I/R) injury (30 min/2 h). Endothelium-dependent vascular relaxation was measured in isolated aortas. Expression of selected proteins in heart tissue was detected by Western blotting. *Results*: QCT reduced systolic blood pressure in both lean and obese 6-month-old rats but had no effect in 1-year-old rats. Diabetes worsened vascular relaxation in both ages. QCT improved vascular relaxation in 6-month-old but worsened in 1-year-old obese rats and had no impact in lean controls of both ages. QCT did not exert cardioprotective effects against I/R injury and even worsened post-ischemic recovery in 1-year-old hearts. QCT up-regulated expression of eNOS in younger and PKCε expression in older rats but did not activate whole PI3K/Akt pathway. *Conclusions*: QCT might be beneficial for vascular function in diabetes type 2; however, increasing age and/or progression of diabetes may confound its vasculoprotective effects. QCT seems to be inefficient in preventing myocardial I/R injury in type 2 diabetes and/or higher age. Impaired activation of PI3K/Akt kinase pathway might be, at least in part, responsible for failing cardioprotection in these subjects.

## 1. Introduction

Quercetin (QCT) is a natural polyphenolic compound widely enriched in human food. Main sources of QCT are different fruits, vegetables and berries, such as green peppers, red onions, elderberries, red apples, and many others [1,2,3]. QCT has been documented to exert various beneficial effects including neuroprotective [4], cardioprotective [3], anticancer [5], antidiabetic [6], as well as antioxidant [7,8], immunomodulatory and anti-inflammatory effects [1,2]. In cardiovascular system, it has been particularly shown to exert antihypertensive [9] and vasorelaxant [10] effects, as well as cardioprotective effects against ischemia-reperfusion (I/R) injury [11,12,13] or anthracycline-induced cardiotoxicity [14,15]. 

Regarding heart-protective effects of QCT, our previous studies demonstrated cardioprotective effects of QCT against I/R injury in isolated perfused rat hearts in both ex vivo [11] and in vivo [12,15] applications. Cardioprotective effects of QCT were further confirmed by other authors in different models of cardiac I/R injury [13,16,17]. Several molecular mechanisms were proposed to be involved in cardioprotective action of QCT including activation of PI3K/Akt pathway, inhibition of apoptosis, up-regulation of connexin-43, activation of endogenous antioxidant enzymes, as well as down-regulation of the HMGB1-TLR4-NF-κB pathway and inhibition of JNK and p38-MAPK pathways [13,15,18,19,20]. QCT has been shown to exert cardioprotective effects against cardiac I/R injury also in streptozotocin-induced diabetic rats, an animal model of diabetes mellitus type 1 [21]. On the other hand, there are no data documenting effect of QCT on cardiac I/R injury in presence of other metabolic comorbidities such as type 2 diabetes or metabolic syndrome. Notably, vast majority of experiments focused on cardiac effects of QCT in I/R models was performed in young animals while most of the patients suffering from ischemia-related pathologies such as ischemic heart disease and acute myocardial infarction are aged individuals with lifestyle-related comorbidities including hypertension, diabetes type 2 or metabolic syndrome. Thus, clinically relevant animal models reflecting age and comorbidities should be used to reveal real therapeutic potential of cardioprotective interventions against I/R including those of QCT.

Regarding vascular effects of QCT, anti-hypertensive effects of QCT have been documented in different animal models of hypertension such as spontaneously hypertensive rats [22,23], *N*-nitro-l-arginine methyl ester (l-NAME)-induced nitric oxide (NO)-deficient hypertension [24], deoxycorticosterone acetate-salt hypertension [25], renovascular hypertension [26], as well as in human studies in hypertensive patients [27,28]. Antihypertensive effect of QCT has been documented also in woman with diabetes type 2 [29] and obese Zucker rats [30]. In addition to blood pressure-lowering effects, QCT has been shown to exert vasorelaxant effects [31,32,33,34] as well as endothelium-protective and anti-atherosclerotic effects [35] in different experimental models, including direct vasoprotective effects in isolated aortas from streptozotocin-induced diabetic rats [36,37,38]. However, effects of QCT on vascular reactivity in diabetes type 2 have still to be explored. Vascular beneficial effects of QCT, particularly its vasorelaxant effects, are most likely mediated through NO-mediated pathways, and thus are in general thought to be endothelium-dependent [31,36,39]. Important role in vasorelaxant effects of QCT may also play K^+^-channels [39,40] and heme oxygenase-1 [35]. However, exact mechanism of QCT vascular effects is still to be clarified.

In line with above mentioned facts, the aims of the present study were: (1) to reveal the cardioprotective potential of chronic application of QCT against I/R injury in isolated perfused hearts from Zucker diabetic fatty (ZDF) rats, an animal model of type 2 diabetes; (2) to explore effects of chronic QCT treatment on blood pressure and endothelium-dependent arterial relaxation in ZDF rats; and (3) to reveal the role of ageing and progression of diabetes in cardiovascular effects of QCT in ZDF rats. Finally, the aim of the study was also to explore mechanisms behind cardiovascular effects of QCT in ZDF rats. To our best knowledge, this is the first study documenting effects of QCT treatment on cardiac I/R injury in diabetes type 2, as well as the first demonstration of chronic QCT effects on vascular reactivity in ZDF rats.

## 2. Results

### 2.1. Effect of QCT on Biometric Parameters and Biochemical Characteristics of Rats 

During the experiment, following parameters have been recorded: body weight (BW), BW gain, food intake, water consumption, and fasting glycaemia. At the end of the experiment, heart weights (HW) were measured. Finally, BW and HW were normalized to tibia length (TL). Lipid profile parameters including total cholesterol, high density lipoprotein-cholesterol, low density lipoprotein-cholesterol, total triglycerides were measured in blood plasma. Biometric and biochemical parameters of rats are summarized in Table 1.

Two-way ANOVA revealed that diabetes significantly increased following biometric parameters in rats of both ages, independently on QCT treatment: BW (*p* < 0.0001 in both ages), BW/TL (*p* < 0.0001 in both ages) and HW/TL (*p* < 0.0001 in 6-month-old; *p* < 0.01 in 1-year-old). Treatment with QCT had no effect on biometric parameters in the study (Table 1). Diabetic animals of both ages were characterized by significantly increased levels of fasting glycaemia as compared to age-matched non-diabetic lean controls, independently on QCT treatment (*p* < 0.01 in 6-month-old; *p* < 0.0001 in 1-year-old). Moreover, fasting glycaemia levels were significantly higher in 1-year-old obese rats as compared to 6-month-old obese rats, independently on QCT treatment (*p* < 0.01), documenting progression of diabetes in time/ageing of ZDF rats. Lean rats of both ages had normal glycaemia levels during whole experiment. QCT did not affect glycaemia levels in all experimental groups (Table 1). In younger (6-month-old) rats, diabetes significantly increased total cholesterol levels (*p* < 0.01) and plasma triglycerides levels (*p* < 0.0001), independently on QCT treatment. All other biochemical parameters were unchanged due to either diabetes or QCT treatment in younger rats. In older (1-year-old) rats, diabetes significantly increased plasma levels of total cholesterol (*p* < 0.0001), triglycerides (*p* < 0.0001), low-density lipoprotein (LDL)-cholesterol (*p* < 0.05) and high-density lipoprotein (HDL)-cholesterol (*p* < 0.0001), independently on QCT treatment. QCT had no effect on biochemical parameters in older rats (Table 1).

### 2.2. Effect of QCT on Blood Pressure 

**Figure 1:**
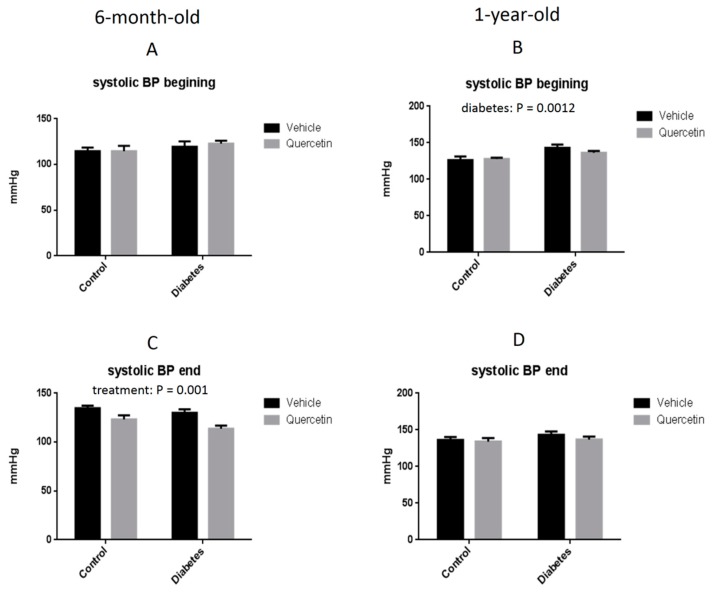
Systolic blood pressure (BP) measured by tail-cuff plethysmography in younger (**A**,**C**) and older (**B**,**D**) ZDF rats: BP beginning—measured before start of quercetin treatment (**A**,**B**); BP end—measured after the completion of quercetin treatment (end of week 6) (**C**,**D**). Results are expressed as means ± SEM. Significant differences were evaluated by two-way ANOVA for main factors diabetes and quercetin treatment.

At the beginning of the experiment (before QCT administration), blood pressure measurements showed no differences in systolic blood pressure among all experimental groups in younger (6-month-old) rats (Figure 1A), but in older rats (1-year-old) there was significantly increased systolic blood pressure in obese rats as compared to lean controls (*p* < 0.01) (Figure 1B). QCT treatment significantly decreased systolic blood pressure in younger rats, independently on the presence of diabetes (*p* < 0.01) (Figure 1C) but had no effect on the systolic blood pressure in older rats (Figure 1D).

### 2.3. Effect of QCT on Vascular Reactivity of Isolated Thoracic Aortas 

**Figure 2:**
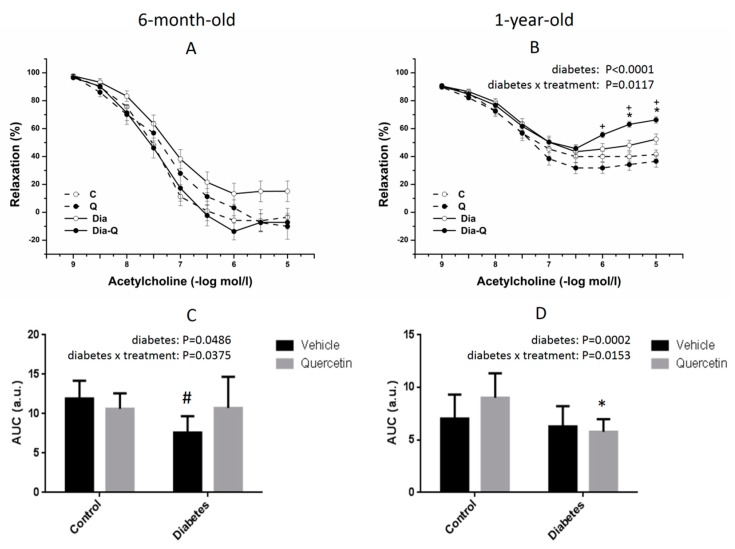
Maximal and overall relaxation of thoracic aorta: diabetes dependence. The endothelium-dependent vasorelaxant responses of thoracic aorta induced by acetylcholine in younger (**A**) and older (**B**) rats; and effect of treatment with quercetin on overall acetylcholine-induced relaxation of thoracic aorta in younger (**C**) and older (**D**) rats. AUC—area under the curve; a.u.—arbitrary units. Results are expressed as mean ± SEM. Significant differences were evaluated by two-way ANOVA for main factors diabetes and quercetin treatment (shown for maximal (**A**,**B**) and overall (**C**,**D**) relaxation). Tukey post hoc test was used to describe the differences in mean values of the experimental groups. # *p* < 0.05 vs. C; * *p* < 0.05 vs. Dia; + *p* < 0.05 vs. Q.

**Figure 3:**
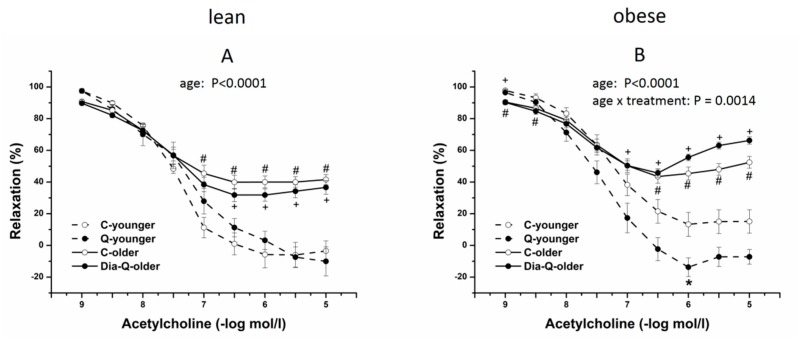
The endothelium-dependent vasorelaxant responses of thoracic aorta induced by acetylcholine in lean (**A**) and obese (**B**) rats. Results are expressed as mean ± SEM. Significant differences were evaluated by two-way ANOVA for main factors diabetes and quercetin treatment (shown for maximal relaxation). Tukey post hoc test was used to describe the differences in mean values of the experimental groups. # *p* < 0.05 vs. C-younger; +*p* < 0.05 vs. Q-younger.

In the first step, we realized the evaluation of differences between responses of rats in different age. Cumulative application of exogenous acetylcholine (10^−9^–10^−5^ mol/L) induced endothelium-dependent-vasorelaxation in phenylephrine (PHE)-precontracted aortic rings. In younger age, there was no significant difference in these responses of lean and obese rats and the treatment with QCT also did not reveal a significant effect neither in lean nor in obese group (Figure 2A). However, AUC (area under the curve) values were significantly lower in obese rats compared to lean group (*p* < 0.05) and there was also confirmed a significant effect of interaction between presence of the obesity and the treatment (*p* < 0.05) (Figure 2C). In older age, there was a significant difference in endothelium-dependent vasorelaxant responses between lean and obese rats (maximum response: *p* < 0.0001), and there was also confirmed a significant effect of interaction between presence of the obesity and the treatment (maximum response: *p* < 0.05, Figure 2B). AUC values were significantly lower in obese rats compared to lean group (*p* < 0.001) and there was also confirmed a significant effect of obesity x treatment interaction (*p* < 0.05, Figure 2D). However, the effect of the interaction between the occurrence of the obesity and treatment with QCT revealed the opposite tendency in younger compared to older rats (Figure 3). 

In the second step, we focused on the evaluation of age-dependencies in the same groups of animals. The maximum endothelium-dependent vasorelaxant response induced by acetylcholine (10^−9^–10^−5^ mol/L) was significantly reduced in groups of older rats compared to younger rats regardless of the occurrence of the obesity (lean rats: *p* < 0.0001; obese rats: *p* < 0.0001, Figure 3). However, whereas there was no significant interaction between age of rats and the treatment with QCT in lean rats (Figure 3A), the significant effect of interaction between age of rats and the treatment with QCT was confirmed in obese rats (*p* < 0.01, Figure 3B). In obese younger rats, the treatment with QCT improved the reduced vasorelaxation, on the other hand, in obese older rats the therapy led to its aggravation.

**Figure 4:**
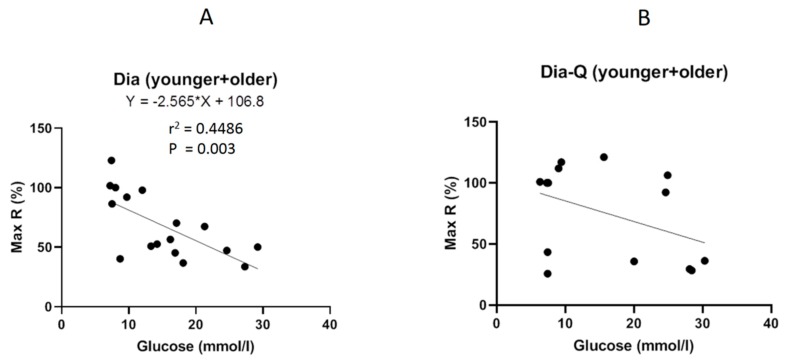
Correlations between maximal relaxation and glycaemia. The mutual relationship between maximal vasorelaxation induced by acetylcholine in thoracic aorta and plasma glucose level in rats without (**A**) and with (**B**) the treatment with quercetin. Max R = maximal relaxation.

We also evaluated the relationship between plasma glucose level and maximal reached endothelium-dependent vasorelaxation. We confirmed that endothelial dysfunction negatively correlated with glucose level regardless of the age (r^2^ = 0.4486, *p* = 0.003; Figure 4A). On the other hand, the effect of QCT seems to be age-dependent because the maximum endothelium-dependent vasorelaxation was not affected by glycaemia (r^2^ = 0.1723, *p* = 0.14; Figure 4B).

### 2.4. Effect of QCT on Post-Ischemic Recovery and Infarct Size in Isolated Hearts

We evaluated effect of QCT on resistance of isolated hearts from ZDF rats to I/R injury by measuring post-ischemic recovery of hemodynamic parameters of isolated Langendorff-perfused hearts in the 40th minute of post-ischemic reperfusion, and by evaluating infarct size in relation to area at risk.

Firstly, there were no significant differences in baseline (pre-ischemic) values of all hemodynamic parameters measured at the end of stabilization period just before initiation of 30-min global ischemia in the hearts from younger rats (6-month-old) (Table 2). In the hearts from older (1-year-old) rats, there were significantly increased left ventricular developed pressure (LVDP) (*p* < 0.001), +(dp/dt)_max_ (*p* < 0.05), −(dp/dt)_max_ (*p* < 0.05) and coronary flow rate (*p* < 0.001) in obese hearts as compared to hearts from age-matched lean controls, while heart rate was significantly decreased (*p* < 0.01) in obese hearts as compared to lean controls (Table 2). Finally, there were no differences in rate-pressure products (RPP, heart rate x LVDP) among the groups before ischemia (Table 2).

**Figure 5:**
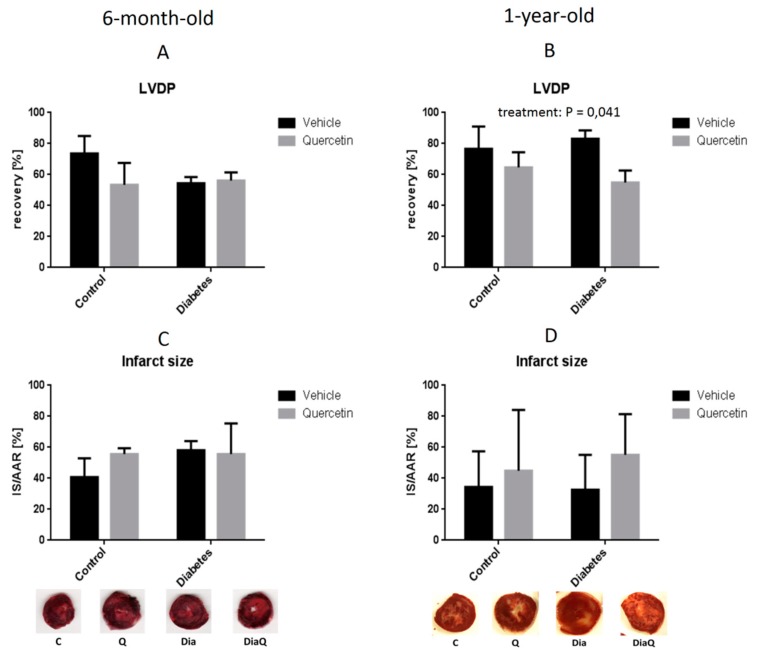
Post-ischemic recovery of left ventricle developed pressure (LVDP, systolic minus diastolic pressure) of isolated hearts from ZDF rats treated with quercetin measured in 40th minute of post-ischemic reperfusion after 30-min global ischemia in younger (**A**) and older (**B**) rats. Infarct size related to area at risk (whole left ventricle in case of global ischemia) measured by 2,3,5-triphenyltetrazolium (TTC) staining after 2-hour reperfusion of isolated hearts from younger (**C**) and older (**D**) ZDF rats. Results are expressed as mean ± SEM. Significant differences were evaluated by two-way ANOVA for main factors diabetes and quercetin treatment.

After ischemia (in the 40th minute of reperfusion), no significant differences were found in post-ischemic recovery of LVDP (Figure 5A), as well as in other hemodynamic parameters including +(dp/dt)_max_, −(dp/dt)_max_, RPP and coronary flow (data not shown) in the hearts from younger rats indicating no effect of either diabetes or QCT treatment on resistance of hearts to I/R. In addition, no significant differences in infarct size were found in the hearts from younger rats (Figure 5C). In older hearts, QCT treatment significantly decreased recovery of LVDP (*p* < 0.05), independently on presence of diabetes (Figure 5B) suggesting detrimental effect of QCT in older hearts. No significant differences were found in recovery of other hemodynamic parameters of older hearts including +(dp/dt)_max_, −(dp/dt)_max_, RPP and coronary flow (data not shown). Finally, infarct size was not influenced by QCT treatment in older rats (Figure 5D). These data indicate that QCT exerts no cardioprotection against I/R-induced development of myocardial infarction in ZDF rats.

### 2.5. Effect of QCT on RISK Pathway Proteins Expression

We evaluated the effect of QCT treatment on the activation of proteins involved in the Reperfusion Injury Salvage Kinases (RISK) pathway. This molecular signaling pathway has been shown to be involved in cardioprotection against I/R in different experimental models of cardiac injury induced by different cardioprotective interventions including QCT-induced cardioprotection in young healthy animals.

**Figure 6:**
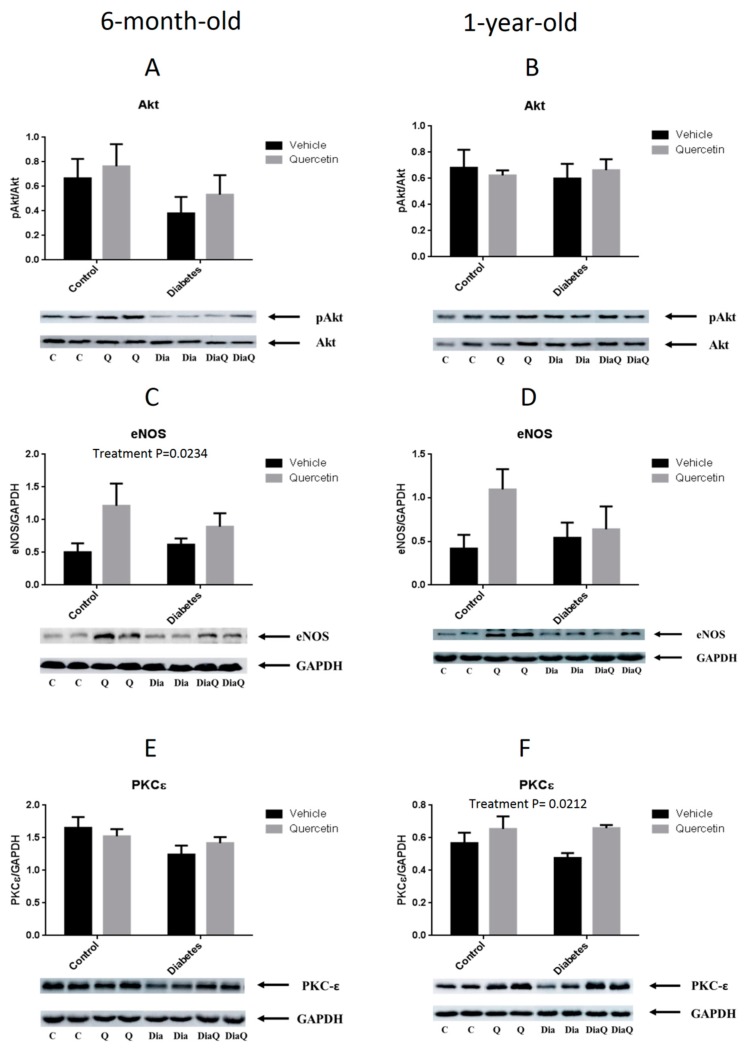
RISK pathway proteins expression in heart tissue Effect of quercetin treatment on proteins involved in the Reperfusion Injury Salvage Kinases (RISK) pathway. Levels and/or specific phosphorylation of proteins were determined by western blotting method using specific antibodies. Akt kinase activation was expressed as a ratio of Akt kinase phosphorylated at Ser473 to protein levels of total Akt kinase in younger (**A**) and older (**B**) ZDF rats. Changes in protein levels of eNOS in younger (**C**) and older (**D**) ZDF rats were normalized to the glyceraldehyde 3-phosphate dehydrogenase (GAPDH) protein levels. PKCε protein levels were determined by specific antibody and obtained data were normalized to the GAPDH in younger (**E**) and older (**F**) ZDF rats. Results are expressed as mean ± SEM. Significant differences were evaluated by two-way ANOVA for main factors diabetes and quercetin treatment.

In younger (6-month-old) ZDF rats, no effect of either diabetes or QCT treatment on Akt kinase activation (expressed as a ratio of Akt kinase phosphorylated at Ser473 to total Akt kinase) was found (Figure 6A). Further, QCT treatment significantly up-regulated eNOS protein expression (*p* < 0.05), independently on the presence/absence of diabetes (Figure 6C). Finally, there were no differences in PKCε protein expression in 6-month-old rats (Figure 6E).

In 1-year-old ZDF rats, there were no significant differences in all three detected proteins of RISK pathway (Akt, eNOS, PKCε) between obese and lean animals, independently on QCT treatment (Figure 6B,D,F). QCT treatment had no effect on Akt phosphorylation (Figure 6B), but significantly increased PKCε protein expression (*p* < 0.05) in heart tissue of treated rats, independently on presence/absence of diabetes (Figure 6F). Thus, Western blotting data showed that QCT partially increased protein levels of some components of RISK pathway in ZDF rats (eNOS in younger and PKCε in older ones); however, the activation of whole RISK pathway have not been detected in the study.

## 3. Discussion

In the present study we have shown that 6 weeks lasting chronic treatment of ZDF rats with QCT reduced blood pressure and improved vascular relaxation in 6-month-old ZDF rats, but had no effect on blood pressure and even worsened vascular relaxation in 1-year-old ZDF rats. Furthermore, QCT treatment had no effect on post-ischemic recovery of functional parameters of isolated hearts from younger rats exposed to I/R, and even worsened post-ischemic recovery of heart function in hearts from older ZDF rat manifested by significantly decreased recovery of LVDP. QCT treatment had no effect on infarct size in younger rat hearts, and even had trend to increase infarct size in older diabetic rat hearts. Finally, QCT partially stimulated the increase of levels of some proteins of RISK pathway (eNOS in younger and PKCε in older ones), but did not influence the activation of whole RISK pathway in either younger or older ZDF rats.

Systolic blood pressure values in both lean and obese untreated ZDF rats correspond to the normal development of this hemodynamic parameter as described previously in other studies [41,42,43]. As expected, obese ZDF rats developed higher blood pressure as compared to their age-matched lean controls. In addition, an age-dependent increase of the systolic blood pressure values was observed in obese rats in our experiment, while blood pressure in lean rats did not increase with age in our study. These results are in concordance with the study of Rivera et al. [30]. In our study, administration of QCT exerted a protective effect on blood pressure in 6-month-old ZDF rats, as we observed a decrease in the systolic blood pressure in both treated groups (Figure 1C). On the other side, 6-week lasting administration of QCT in 1-year-old lean and obese rats had no significant effect on blood pressure. In our previous study we found that prolonged administration of QCT failed to modulate blood pressure also in 20-week-old Wistar rats [15]. On the other side, QCT was able to prevent blood pressure increase induced by chronic treatment with doxorubicin. Together, it seems that modulation of blood pressure by QCT might be influenced by age, metabolic status as well as eventual co-treatments.

The systolic blood pressure values largely reflected the resultant endothelium-dependent vascular reactivity of the aorta. In 6-month-old ZDF rats of both genotypes, but not in 1-year-old, we observed an improvement in the vascular reactivity after QCT application. This is in concordance with the study of Rivera et al. [30] that showed that the chronic administration of QCT enhances aortic eNOS expression in obese ZDF rats, which is directly related to the decrease in blood pressure as the result of improved NO bioavailability. It was shown that impaired vascular reactivity in Zucker rats and decreased activity of eNOS/NO bioavailability in the vascular endothelium is associated with increased superoxide production as the result of hyperglycemia [44]. Accordingly, other authors obtained similar results, e.g., in spontaneously hypertensive rats where systolic blood pressure decreased by 15% after 12-weeks [45] or 5-weeks of QCT administration as a result of improved endothelium-dependent vascular reactivity of the aorta [22,46], or in streptozotocin-induced diabetes in Wistar-Kyoto rats [47].

As seen from the presented results on isolated thoracic aortas, elevated glycaemia in obese ZDF correlated with the degree of reduction in endothelium-dependent relaxant response induced by acetylcholine. This impairment was clearly manifested in younger, 6-month-old obese ZDF, which had significantly decreased acetylcholine-induced relaxation in thoracic aortas when compared to their age-matched lean controls. However, in older, 1-year-old group, the adverse effect of aging on endothelial function strongly prevailed in obese as well as in lean rats, and the relaxant responses markedly decreased to the similar level in both, comparing to their respective younger groups. This is in accordance with the well-known age-related negative alterations in arterial function in general, as described by other authors [48,49,50].

There is a large volume of published studies describing the beneficial effect of QCT on cardiovascular system which is often associated with its potentiating influence on arterial relaxation. This was confirmed in many experimental models of hypertension [51,52,53] and diabetes [54,55] in which treatment with QCT led to blood pressure decrease, glycaemia improvement, and enhancement of arterial endothelium-dependent relaxation. In the present study, interestingly, the character of effect of QCT on arterial relaxation in obese rats was dependent on their age. The ameliorating action of QCT on arterial relaxation was evident only in 6-month-old obese ZDF and was accompanied by blood pressure decrease in these rats. However, such effect was not detected in 1-year-old obese ZDF, in which QCT showed tendency to worsen the relaxant responses of thoracic aorta, without any noticeable effect on blood pressure. In thoracic aortas of this older group of obese rats treated with QCT, the dose-dependent relaxant responses were markedly reversed at higher concentrations of acetylcholine. Although the endothelium-dependent vasorelaxation was not affected by glycaemia, the effect of QCT seems to be not only age-dependent. Aggravating effect of QCT treatment on endothelial function was not found in old lean rats in spite of noticeable impairment of aortal relaxation due to aging indicating possible release of endothelium-derived contracting factors which is often found in arteries from hypertensive or diabetic rats [50,56]. The present results indicate that the mechanism of age-related impairment of arterial relaxant responses might be different in obese and in lean Zucker rats. These findings also demonstrate that the endothelium of old obese ZDF could have a potential of release of contracting factors which may stay latent in certain conditions, or may be provoked by the action of some pharmacological stimuli.

It has been shown previously that QCT exerts cardioprotective effects in different models of myocardial I/R injury in young healthy animals [13,16,17]. In our recent studies, we have demonstrated cardioprotective effects of QCT against I/R injury in both acute in vitro [11] and chronic in vivo administration [12,15]. However, for successful prediction of QCT effectivity in preventing cardiac I/R injury in humans, it should be tested in clinically relevant animal models of myocardial I/R injury reflecting age, comorbidities and eventually co-medications occurring in real human patients suffering from major ischemic diseases including coronary heart disease and myocardial infarction. In line with this need, we explored effects of QCT on myocardial I/R injury in animals suffering from a major metabolic comorbidity present in humans, diabetes type 2, using ZDF rats in the current study. Moreover, rats in our study were older than usually used young adults (3-month-old) in majority of experimental studies, as we used 6-month-old and 1-year-old ZDF rats that better reflect real age of human patients suffering from cardiovascular disease and diabetes type 2. However, even older rats used in our study (1-year-old) are still age-equal to only 30-year-old humans [57]. We have shown that QCT did not prevent I/R injury in ZDF rat hearts of both ages as demonstrated by post-ischemic recovery of heart function and infarct size post I/R. Moreover, in older rat hearts, QCT even exacerbated I/R injury as demonstrated by significantly decreased recovery of LVDP and trend to increase infarct size as compared to non-treated animals. When compared to previous studies in young healthy animals, and when compared effects of QCT in younger versus older ZDF rats in the present study, our data suggest that progression of diabetes (including increasing glycaemia levels) and/or ageing may contribute to abolished cardioprotective effects of QCT against myocardial I/R injury in ZDF rats. Nevertheless, based on the results of current study it is impossible to identify the main factor contributing to failure of QCT to confer cardioprotection against I/R. Since Annapurna et al. [21] found that QCT exerted cardioprotective effect against I/R injury in type 1 streptozotocin-induced diabetic rat hearts similar to that obtained in non-diabetic healthy rats, it might be speculated that diabetes/increased glycaemia is not the main confounding factor for cardioprotection by QCT, and that age may play a key role. This conclusion is supported by the data obtained in the current study where vascular relaxation was improved by QCT in younger but worsened in older rats, and no correlation between vascular relaxation and glycaemia levels was found in QCT treated animals. Also, our previous study exploring effects of QCT on cardiac I/R injury in rats in different age have shown that age may play an important role in cardioprotective effects of QCT [12]. In Wistar rats we found that QCT protected hearts against I/R injury in juvenile rats but not in adult rats. Moreover, increasing age has been identified as confounding factor for cardioprotection of other cardioprotective interventions, e.g., ischemic preconditioning [58]. It should be also pointed out that QCT did not prevent hearts from I/R injury even in lean controls in both ages of rats in the present study, what supports the view that elevated glycaemia is probably not the major confounding factor for cardioprotective effects QCT. Other factors like age or animal strain might affect cardioprotective potential of QCT to prevent cardiac I/R injury which seemed very promising from previous studies in young animals. Different metabolic conditions in ZDF rats as compared to metabolically standard animal strains, e.g., Wistar rats used in our previous studies, might also influence the metabolism of QCT in gastrointestinal tract and this may also be contributing factor to different effects of QCT in ZDF rats as compared to its effects in Wistar rats in other studies. Thus, additional studies are needed to identify the main confounding factor for cardioprotection by QCT.

Cardioprotective effects of QCT and its derivatives have been associated with different molecular changes including activation of PI3K/Akt pathway [15,20,59] which is a part of so called RISK (Reperfusion Injury Salvage Kinases) pathway [60]. RISK pathway was found to be involved in cardioprotection against I/R injury achieved by different interventions, e.g., ischemic conditioning [61] or oxytocin-induced cardioprotection [62] in our previous studies. In the present study, we have shown that some proteins of RISK pathway are modulated in ZDF rats due to QCT treatment, particularly up-regulated eNOS in 6-month-old rats and up-regulated PKCε in 1-year-old rats. However, the overall activation of RISK pathway has not been detected in the present study, suggesting that impaired or insufficiently activated PI3K/Akt signaling as a part of RISK pathway might be, at least in part, the reason for failure of QCT to protect hearts against I/R injury in ZDF rats. This view is supported by previous findings where cardioprotection against I/R by other interventions were associated with impaired PI3K/Akt pathway, e.g., failure to precondition rat hearts against I/R under conditions of simulated hyperglycemia [63]. It should be mentioned that other mechanisms found to be involved in the effects of QCT, which have not been evaluated in the present study, might also be influenced by age or hypermetabolic state, and thus contribute to failing cardioprotection by QCT in diabetic hearts. For example, our recent data point to possible important role of matrix metalloproteinases (MMPs) in realization of effects induced by QCT. In doxorubicin-treated rats QCT increased myocardial resistance to I/R injury and cardioprotective effects of QCT were associated with prevention of MMP activation [15]. In the same study we have also reported that activation of connexin-43 and inhibition of apoptosis are involved in cardioprotective effects of QCT. Thus, revealing impact of age and diabetes on additional molecular pathways like MMPs, apoptosis or gap junction proteins might help to uncover the reason of failed cardioprotection by QCT in ZDF rats.

## 4. Materials and Methods

### 4.1. Materials

**Figure 7:**
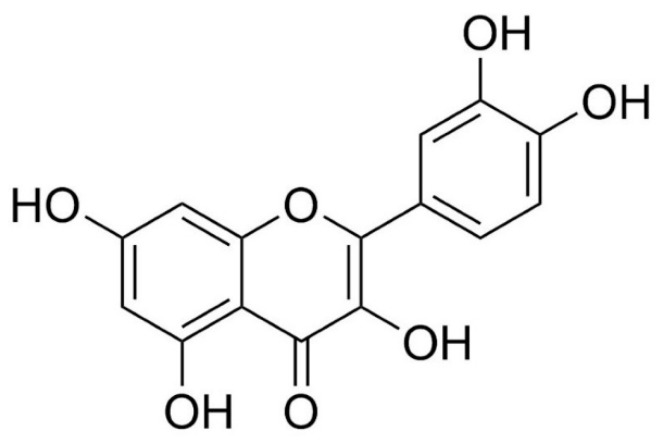
Chemical structure of quercetin (3,5,7,3′,4′-pentahydroxyflavone).

Quercetin (QCT, Figure 7) was purchased from Sigma Aldrich (cat. No Q4951, St. Louis, MO, USA). Primary antibodies against Akt kinase phosphorylated at serine 473 (p-Akt, #4058) and peroxidase-labeled anti-rabbit or anti-mouse secondary antibodies were purchased from Cell Signaling Technology (Danvers, MA, USA). Antibodies against total Akt kinase (sc-8312), PKCε (sc-214), eNOS (sc-653), and GAPDH (sc-32233) were obtained from Santa Cruz Biotechnology (Santa Cruz, CA, USA).

### 4.2. Experimental Model

Obese Zucker diabetic fatty (ZDF) rats (fa/fa) and their age-matched non-diabetic lean controls (fa/+) were used in the study. Animals of two ages, 6-month-old and 1-year-old (at the beginning of the experiment), were used to reveal the role of ageing and progression of diabetes in cardiovascular effects of QCT. All animals were housed at a stable temperature of 22 ± 2 °C and humidity of 45–65%. Rats were fed with normal chow KZ-P/M (complete feed mixture for rats and mouse, reg. no 6147, Dobra Voda, Slovak Republic) and allowed access to drinking water ad libitum. The feed and water consumption as well as body weight and water intake were monitored 3–4 times per week (every second day). Rats of both ages were divided into four experimental groups (n = 12–17 in each group, Table 3): lean vehicle-treated controls (C), lean QCT-treated (Q), obese diabetic vehicle-treated (Dia) and obese diabetic QCT-treated (DiaQ). In the Q and DiaQ groups, the rats received QCT in the dose of 20 mg/kg/day during 6 weeks. QCT was dissolved in small amount of ethanol and served on a piece of biscuit (vehicle) as described previously [15].

**Figure 8:**
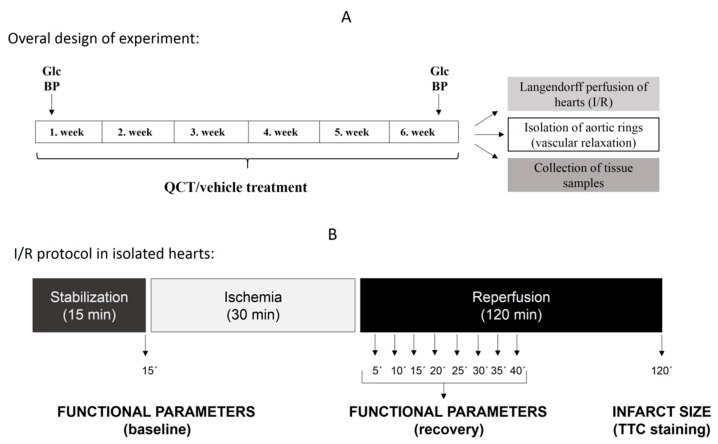
Overall experimental design (**A**) and experimental protocol of I/R in isolated hearts (**B**). Abbreviations: Glc, fasting glycaemia measurements; BP, blood pressure measurements.

During experiment, fasting glycaemia (measured in blood drop from rat tail vein by glucometer) and blood pressure were measured 2 times: before beginning of QCT treatment and at the end of QCT treatment (end of week 6). The overall experimental protocol is summarized in Figure 8. 

After the completion of QCT treatment, animals were anaesthetized with thiopental (50 mg/kg, i.p.) and heparinized (500 IU, s.c.); excised hearts were either perfused according to Langendorff and tested on I/R injury or used for tissue sample analyses. Isolated arteries were used either for vascular reactivity measurements or stored for further tissue analyses. Arterial blood taken from abdominal aortas before heart excision was collected in anticoagulant tubes (FL Medical, Torreglia (Padova), Italy); blood plasma obtained by centrifugation was stored for further analyses at −70 °C. All animal experiments were performed in accordance with the rules issued by the State Veterinary Administration of the Slovak Republic, legislation No 377/2012 and with the regulations of the Animal Research and Care Committee of Centre of Experimental Medicine SAS—Project no. 2237/18-221/3, approved on 21 August 2018.

### 4.3. Blood Pressure (BP) and Heart Rate (HR) Measurement

The systolic and diastolic blood pressure and heart rate (HR) were measured by the non-invasive method of tail cuff plethysmography and the data were recorded by PowerLab 4/30 (ADInstruments, Budapest, Hungary) and evaluated as described previously [64]. Animals were exposed to handling before measurements to avoid stress-induced BP elevation during the procedure. BP data were obtained from on average five measurements of the same animal at one time. The BP measurements were performed two times during whole experiment: before the first QCT or vehicle application (beginning of week 1), and after the end of QCT/vehicle application (end of week 6).

### 4.4. Perfusion Technique and Determination of Heart Function

Isolated heart protocols were performed as described in our previous studies [11,12,15,65,66]. Briefly, after rapid thoracotomy, rat hearts were excised and immediately placed in ice-cold perfusion buffer, cannulated via the aorta and placed onto Langendorff set-up. Hearts were perfused at a constant perfusion pressure of 73 mmHg at 37 °C. Modified Krebs–Henseleit buffer gassed with 95% O_2_ and 5% CO_2_ (pH = 7.4) containing (in mmol/L): NaCl 118.0; KCl 3.2; MgSO_4_ 1.2; NaHCO_3_ 25.0; KH_2_PO_4_ 1.18; CaCl_2_ 2.5; glucose 7.0.; was used as perfusion solution. Solution was filtered through 1.2 μm porosity filter (Merck Millipore, Burlington, MA, USA) to remove contaminants. An epicardial electrocardiogram was registered by means of two stainless steel electrodes attached to the apex of the heart and aortic cannula. Left ventricular pressure was measured by a water-filled elastic balloon inserted into the left ventricle via the left atrium (adjusted to obtain end-diastolic pressure of 1–5 mmHg before ischemia) and connected to a pressure transducer (4/30, ADInstruments). Left ventricular developed pressure (LVDP, systolic minus diastolic pressure), maximal rates of pressure development and fall, +(dP/dt)_max_ and −(dP/dt)_max_ as the indexes of contraction and relaxation, heart rate (calculated from ECG), and coronary flow were measured to assess cardiac function using PowerLab Chart 7 software (ADInstruments). Global ischemia was maintained for 30 min, followed by 120 min of reperfusion. Functional parameters of hearts were measured during initial 40 min of reperfusion. Recovery of functional parameters after ischemia was expressed as a percentage of pre-ischemic baseline values. At the end of reperfusion, infarct sizes were visualized histochemically using triphenyltetrazolium chloride (TTC)-staining method.

### 4.5. Infarct Size Determination

At the end of reperfusion, the hearts were stained with 1% 2,3,5-triphenyltetrazolium chloride (TTC) (Sigma-Aldrich, St. Louis, MO, USA) dissolved in 0.1 M phosphate buffer (pH = 7.4) to determine infarction size as described previously [11]. The hearts were then stored overnight in 10% neutral formaldehyde solution and cut perpendicularly to the long axis of the ventricle into the 1 mm thick slices. The infarct size and the size of area at risk were measured by a computerized planimetric method. The infarct size was normalized to the size of area at risk, which in the model of global ischemia was the whole area of left ventricle.

### 4.6. Vascular Reactivity Measurements

Vascular reactivity was measured in isolated thoracic aorta as described previously [67]. Briefly, ring preparations (5 mm length) of isolated thoracic aorta were vertically fixed between 2 stainless wire triangles and immersed in 20 mL incubation organ bath with Krebs solution containing (in mmol/L): NaCl 118, KCl 5, NaHCO_3_ 25, MgSO_4_·7H_2_O 1.2, KH_2_PO_4_ 1.2, CaCl_2_ 2.5, glucose 11, CaNa_2_EDTA 0.032. This solution was oxygenated with 95% O_2_ and 5% CO_2_ mixture and kept at 37 °C. The triangles were connected to sensors of the isometric tension (FSG-01, MDE, Budapest, Hungary), and the changes in tension were registered by an AD converter NI USB-6221 (National Instruments, Austin, TX, USA; MDE, Budapest, Hungary) and registered by SPEL Advanced Kymograph (MDE) software. The resting tension of 1 g was applied to each ring and maintained throughout a 45 to 60 min of equilibration period until the stress relaxation no longer occurred.

Aortic preparations were pre-contracted with a single dose of phenylephrine (PHE; 10^−6^ mol/L) applied into incubation Krebs solution. After reaching the steady state, acetylcholine was applied cumulatively (10^−10^–10^−5^ mol/L) to induce dose-dependent relaxant responses. The magnitude of PHE-induced pre-contraction was measured in mN. The rate of relaxation was expressed as a percentage of the PHE-induced contraction. Area under curve (AUC, in arbitrary units) was calculated from individual concentration-response curves in each experimental group, using absolute values of arterial tension in mN normalized to the length of respective ring preparation (in mm). Endothelial function was evaluated depending of obesity or age and the relationship between maximal vasorelaxant response and plasma glucose level was also determined using linear regression analysis.

### 4.7. Samples Collection

Excised hearts were weighted and separated to the atria, septum and left and right ventricles. Heart weights (HW) were registered and normalized to tibia length. Collected heart tissue samples were immediately frozen in liquid nitrogen and stored at −70 °C until used for biochemical analyses. The plasma samples were prepared from blood drawn from abdominal aorta before excision of the heart. Blood samples were immediately added to the anticoagulation tubes (FL Medical), followed by centrifugation for 10 min at 1200× *g* to obtain plasma samples. The prepared plasma samples were stored at −70 °C until further analyses.

### 4.8. Preparation of Tissue Protein Fractions and Western Blot (WB) Analysis

The tissue samples were re-suspended in ice-cold homogenization buffer containing (in mmol/L): 50 Tris-HCl, 250 sucrose, 1.0 EGTA, 1.0 dithiothreitol, 1.0 phenylmethylsulfonyl fluoride and 0.5 sodium orthovanadate (pH 7.4) and homogenized with a Teflon homogenizer. The homogenates were centrifuged at 800× *g* for 5 min at 4 °C, pellets were discarded after centrifugation and supernatants were centrifuged again at 16,100× *g* for 30 min. Supernatants after the second centrifugation called “soluble fraction” were stored and used for WB analysis of cytosolic proteins. Pellets after second centrifugation were re-suspended in homogenization buffer containing (in mmol/L): 50 mM Tris-HCl, 250 sucrose, 1.0 EGTA, 1.0 dithiothreitol, and 0.2% Triton X-100. Prepared samples were centrifuged at 16,100× *g* for 5 min. Protein concentrations were estimated by the Bradford method [68]. For WB analysis, samples containing equivalent amounts of proteins per lane were separated by sodium dodecyl sulfate (SDS)-polyacrylamide gel electrophoresis. After electrophoretic separation, the proteins were transferred onto nitrocellulose membranes, and after blocking of non-specific binding sites with 3% non-fat milk, the membranes were overnight incubated with primary antibodies at 4 °C. Membranes were then incubated with corresponding peroxidase-labeled secondary antibodies. Peroxidase reactions were detected by the enhanced chemiluminescence method using the Amersham Imager 600 (Amersham (GE Healthcare), Chicago, ILL, USA). Densitometric quantification of protein levels was performed using the ImageJ software.

### 4.9. Biochemical Evaluations in Blood Plasma

The concentrations of total cholesterol, LDL-cholesterol, HDL-cholesterol, triglycerides and total protein in plasma were determined by the use of BioLis 24i/CLC480 Chemistry Analyzer (Carolina Liquid Chemistries, Greensboro, NC, USA).

### 4.10. Statistical Evaluation

Results are expressed as means ± standard errors of means (SEM). Two-way analysis of variance (ANOVA) with main factors: obesity x treatment or age x treatment, respectively, followed by Tukey post hoc test, were used to analyze differences in mean values of the experimental groups. Differences were considered significant at *p* < 0.05.

## 5. Conclusions

Based on the results of the present study, it could be concluded that QCT might be potentially beneficial for vascular function and in preventing hypertension in individuals suffering from diabetes type 2; however, increasing age may negatively influence vascular beneficial effects of QCT. On the other hand, QCT seems to be inefficient in preventing myocardial I/R injury in subjects suffering from diabetes type 2 and/or in individuals in higher age. Thus, the presence of metabolic comorbidities and age of patients should be taken into consideration when potentially using QCT for prevention and/or treatment of cardiovascular disease in humans.

In general, results of our study clearly demonstrated that confounding factors like age or hypermetabolic state may significantly influence the beneficial effects of natural substances, e.g., polyphenols (including QCT), on the cardiovascular health. Thus, our study opened possible future directions for cardiovascular research reflecting an urgent need to test beneficial effects of any substances aimed to improve cardiovascular health in the presence of potential confounding factors, not only age and metabolic disorders, but also other factors such as sex, lifestyle-related negative factors (smoking, obesity, alcohol consumption), mental disorders, and others.

## Figures and Tables

**Table 1:** Biometric and biochemical parameters of 6-month-old and 1-year-old ZDF rats.

	6-Month-Old Rats							1-Year-Old Rats						
	LEAN		OBESE		Two-Way ANOVA			LEAN		OBESE		Two-Way ANOVA		
	C	Q	Dia	DiaQ	Diabetes	Treatment	Interaction	C	Q	Dia	DiaQ	Diabetes	Treatment	Interaction
**BW (g)**	349 ± 7	346 ± 9	493 ± 8	487 ± 10	****	ns.	ns.	406 ± 10	413 ± 8	520 ± 25	514 ± 24	****	ns.	ns.
**BW/tibia (g/mm)**	9.2 ±0.2	8.9 ± 0.2	13.2 ± 0.2	12.9 ± 0.3	****	ns.	ns.	10.2 ± 0.2	10.3 ± 0.2	13.7 ± 0.6	13.4 ± 0.7	****	ns.	ns.
**HW/tibia (g/mm)**	0.03 ± 0.001	0.03 ± 0.001	0.04 ± 0.001	0.04 ± 0.001	****	ns.	ns.	0.03 ± 0.001	0.03 ± 0.001	0.04 ± 0.001	0.04 ± 0.01	**	ns.	ns.
**Glucose (mmol/L)**	7.3 ± 0.4	6.9 ± 0.2	11.2 ± 1.3	12.6 ± 1.9	**	ns.	ns.	6.1 ± 0.1	5.9 ± 0.2	16.9 ± 1.6	18.5 ± 2.1	****	ns.	ns.
**TAG (mmol/L)**	0.38 ± 0.13	0.40 ± 0.08	5.87 ± 0.89	5.76 ± 1.23	****	ns.	ns.	0.24 ± 0.05	0.18 ± 0.03	3.66 ± 0.41	3.67 ± 0.39	****	ns.	ns.
**Ch. (mmol/L)**	1.92 ± 0.09	1.63 ± 0.07	2.44 ± 0.28	2.7 ± 0.32	**	ns.	ns.	2.85 ± 0.11	2.81 ± 0.08	4.86 ± 0.41	4.79 ± 0.25	****	ns.	ns.
**HDL-Ch. (mmol/L)**	0.93 ± 0.08	0.67 ± 0.07	1.01 ± 0.18	0.97 ± 0.16	ns.	ns.	ns.	1.36 ± 0.04	1.37 ± 0.04	2.36 ± 0.13	2.37 ± 0.07	****	ns.	ns.
**LDL-Ch. (mmol/L)**	0.46 ± 0.03	0.36 ± 0.02	0.34 ± 0.07	0.32 ± 0.06	ns.	ns.	ns.	0.82 ± 0.04	0.81 ± 0.02	1.05 ± 0.08	0.91 ± 0.07	*	ns.	ns.

Experimental groups: C—non-treated lean controls; Q—quercetin-treated lean rats; Dia—non-treated obese diabetic rats; DiaQ—quercetin-treated obese diabetic rats; Abbreviations: BW—body weight; HW—heart weight; TAG—triacylglycerides; Ch—cholesterol; HDL—high-density lipoprotein; LDL—low-density lipoprotein. Data are presented as means ± SEM. Significant differences were evaluated by two-way ANOVA with main factors diabetes and quercetin treatment. * *p* < 0.05; ** *p* < 0.01; **** *p* < 0.0001.

**Table 2:** Pre-ischemic (baseline) values of functional parameters of isolated hearts.

	6-Month-Old Rats							1-Year-Old Rats						
	LEAN		OBESE		Two-Way ANOVA			LEAN		OBESE		Two-Way ANOVA		
	C	Q	Dia	DiaQ	Diabetes	Treatment	Interaction	C	Q	Dia	DiaQ	Diabetes	Treatment	Interaction
**LVDP (mmHg)**	81 ± 7	72 ± 9	83 ± 8	71 ± 3	ns.	ns.	ns.	73 ± 5	90 ± 9	114 ± 9	109 ± 4	***	ns.	ns.
**+(dp/dt)_max_ (mmHg)**	1789 ± 179	1548 ± 241	1615 ±187	1383 ± 123	ns.	ns.	ns.	1522 ± 135	1909 ± 213	2143 ± 156	2048 ± 110	*	ns.	ns.
**−(dp/dt)_max_ (mmHg)**	1287 ± 127	1187 ± 183	1317 ±154	1136 ± 112	ns.	ns.	ns.	1158 ± 143	1451 ± 198	1690 ± 128	1661 ± 72	*	ns.	ns.
**HR (beats/min)**	226 ± 28	237 ± 13	207 ± 18	173 ± 19	ns.	ns.	ns.	227 ± 29	210 ± 10	154 ± 12	162 ± 17	**	ns.	ns.
**RPP (mmHg/min)**	18,583 ± 3047	16,867 ± 2304	17,495 ± 2975	12,300 ± 1629	ns.	ns.	ns.	16,533 ± 2298	19,060 ± 2675	17,341 ± 1494	17,794 ± 2107	ns.	ns.	ns.
**CF (mL)**	12 ± 2	16 ± 2	15 ± 1	12 ± 2	ns.	ns.	ns.	12 ± 2	12 ± 0	18 ± 1	16 ± 1	***	ns.	ns.

*Experimental groups*: C—non-treated lean controls; Q—quercetin-treated lean rats; Dia—non-treated obese diabetic rats; DiaQ—quercetin-treated obese diabetic rats; *Abbreviations*: LVDP—left ventricular developed pressure; ±(dp/dt)_max_—maximal rate of contraction and relaxation of left ventricular pressure (contraction/relaxation index); HR—heart rate; RPP—rate pressure product (LVDPxHR); CF—coronary flow. Data are presented as means ± SEM. Significant differences were evaluated by two-way ANOVA with main factors diabetes and quercetin treatment. * *p* < 0.05; ** *p* < 0.01; *** *p* < 0.001.

**Table 3:** Summary of the number of animals (rats) in experimental groups.

Experimental Group	6-Month-Old	1-Year-Old
Control (C)	n = 12	n = 12
QCT-treated (Q)	n = 12	n = 13
Diabetic (Dia)	n = 12	n = 16
Diabetic QCT-treated (DiaQ)	n = 12	n = 17

## Supplementary Files

Supplementary File 1
