# Supplementary material for: Quercetin Exerts Age-Dependent Beneficial Effects on Blood Pressure and Vascular Function, But Is Inefficient in Preventing Myocardial Ischemia-Reperfusion Injury in Zucker Diabetic Fatty Rats
Source: Molecules. 2020 Jan 2;25(1):187. doi: 10.3390/molecules25010187 (PMC6983107; doi:10.3390/molecules25010187)
Supplement: Supplementary File 1 [file molecules-25-00187-s001.pdf]

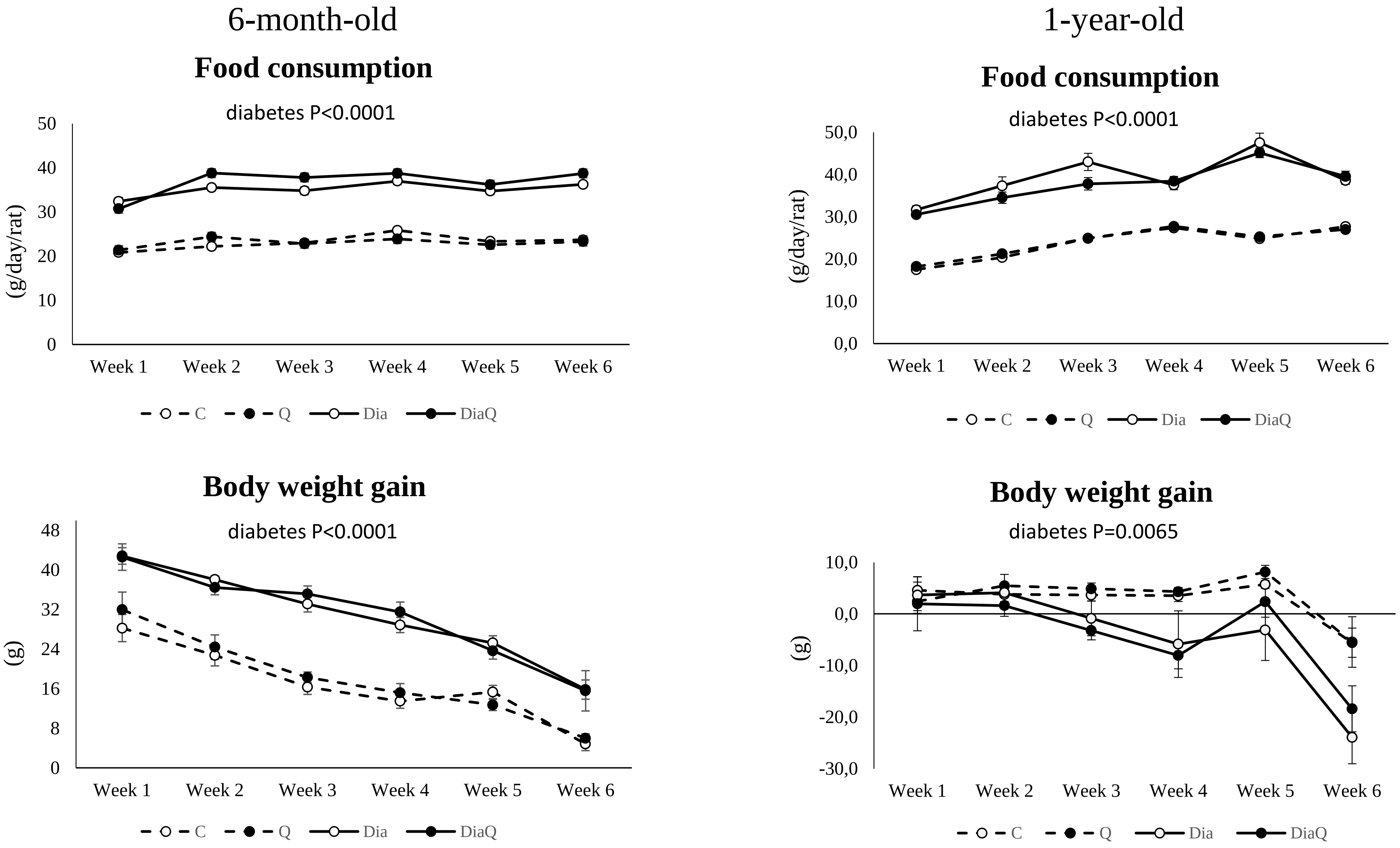

Supplementary Figure. Average daily food consumption by weeks (upper graphs), and weekly body weight gain (lower graphs) in ZDF rats treated with quercetin. Results are expressed as mean ± SEM. Significant differences were evaluated by two-way ANOVA for main factors diabetes and quercetin treatment (significancies are shown for average daily food consumption (upper) and total body weight gain (lower)).
